# Learning from errors and failure in educational contexts: New insights and future directions for research and practice

**DOI:** 10.1111/bjep.12716

**Published:** 2024-09-24

**Authors:** Susanne Narciss, Ecenaz Alemdag

**Affiliations:** ^1^ Psychology of Learning and Instruction Technische Universität Dresden Germany; ^2^ Center of Tactile Internet with Human in the Loop (CeTI) Technische Universität Dresden Dresden Germany

**Keywords:** erroneous example, error climate, instructional support, learning from errors, learning from failure

## Abstract

**Background and Aims:**

Although errors and failures are indispensable parts of the learning process, the current theoretical models and empirical research remain inadequate to provide a comprehensive perspective for learning from errors, considering the roles of different agents, settings, and support mechanisms. Addressing these gaps in the literature, this special issue collects 11 research papers related to learning from errors and failure in educational contexts. In this commentary, we synthesize the findings of these papers with previous work, address conceptual and methodological challenges based on these papers and their implications, and provide suggestions to enhance educational practices.

**Results:**

The special issue papers varying in regard to research design, subject domain, participants, and learning setting presented findings about four main themes: (1) contextual factors (e.g. organization and error climate) as an enabler and barrier, (2) individual factors (e.g. motivational beliefs and emotions) in learners' processing of errors, (3) error‐ or failure‐related learning processes, and (4) instructional strategies (e.g. feedback and prompts) to support learning from errors. Critical evaluation of these papers also revealed conceptual (e.g. error vs. failure) and methodological (e.g. generic vs. error‐specific measures and instruments) challenges, which also paved the way for directions for future research.

**Conclusion:**

Overall, 11 papers in this special issue provide significant empirical evidence about learning from errors and failure in educational contexts. Synthesizing the findings of both these papers and prior research, we also present suggestions to construct an educational environment conducive to learning from errors.

## INTRODUCTION

Errors and failure experiences can profoundly influence the further stages of learning. The studies presented in this special issue reveal that research on learning from errors in educational contexts is a broad and complex field of research. The studies used various theoretical perspectives and methodological approaches, focused on different individual, situational, and contextual factors, and, by doing so, illustrated how important questions concerning learning from errors can be examined in diverse ecological learning contexts. Furthermore, they showcase how, based on various theoretical perspectives, diverse methodological approaches can be used to systematically examine under what conditions errors and/or failures can unfold their benefits for learning or have detrimental effects. In our discussion, we will first provide a synthesis of the major research findings and link them to findings and issues addressed by prior work on learning from errors. Second, we will discuss selected conceptual challenges and issues raised by the studies for further research. Third, we will provide an overview of the methodological approaches used by the studies in the special issue and discuss methodological challenges and their implications. Finally, we will outline some practical implications in order to facilitate the transfer of scientific insights obtained through this special issue and prior studies into educational practice.

## SYNTHESIS OF THE SPECIAL ISSUE PAPERS WITH PREVIOUS WORK

The 11 papers of this special issue provide important new insights on how various (a) contextual factors, (b) individual factors, as well as their interplay with (c) error‐ or failure‐related learning processes, and (d) instructional strategies affect the learning from errors and/or failures in educational contexts (for an overview see Table 1).

**TABLE 1:** Overview of major issues addressed by the special issue studies and other research on learning from errors and failures.

Issue	Category	Examples	Studies special issue
Contextual factors	Interpersonal aspect	Error climate; shared understanding of task goals, values, and strategies	Steuer et al. (2024); Dresel et al. (2024); Peterson et al. (2024)
	Teacher/educator attitudes	Failure mindsets; stereotypes	Di Battista (2024); Simpson et al. (2023)
	Learning context	Formal vs. informal, high vs. low‐stake exams	Across studies of the special issue
	Task characteristics	Complexity and content of tasks	Schmid et al. (2024)
Individual factors	Demographics	Age; grade; gender	Dresel et al. (2024)
	Motivational beliefs and emotions	Goal orientation; self‐concept; fear of failure; anxiety	Schmidt et al. (2024); Peterson et al. (2024)
	Error‐related attitudes	Error‐related beliefs; error‐learning orientation; failure mindsets	Tulis and Dresel (2024); Schmid et al. (2024); Simpson et al. (2023)
	Regulation styles	Emotion regulation styles	Sharabi & Roth (2024)
	Knowledge	Domain knowledge; task knowledge; strategy knowledge	
	Metacognition	Response confidence	
Error‐ or failure‐related learning processes	Cognitive	Activating prior knowledge, comparing with reference information	
	Metacognitive	Reflections on reasons of errors and their corrections; change of strategies	Metcalfe et al. (2024); Tulis and Dresel (2024)
	Motivational	Self‐efficacy; persistence; goal‐directedness	DeLiema et al. (2024; Tulis and Dresel (2024)
	Emotional, affective	Frustration, surprise, confusion, joy, boredom, fear	DeLiema et al. (2024); Dresel et al. (2024); Schmid et al. (2024); Soncini et al. (2024)
	Behavioural	Persisting, giving up, help‐seeking; error correction; avoiding recurring errors; adaptive vs. maladaptive coping practices	DeLiema et al. (2024); Dresel et al. (2024); Soncini et al. (2024); Sharabi & Roth (2024); Tulis and Dresel (2024)
	Social	Negotiating with peers and teachers; conversation among mother and child	DeLiema et al. (2024); Peterson et al. (2024)
Instructional strategies to support learning from errors	Teaching strategies	Direct instruction; interactive instruction	Metcalfe et al. (2024)
	Feedback	Motivational, elaborated, interactive, delayed feedback	Metcalfe et al. (2024); Soncini et al. (2024)
	Instructional prompts	Cognitive, motivational, and metacognitive prompts	Tulis and Dresel (2024); Schmid et al. (2024)
	Training	Error‐management training, professional development programs for teachers; workshops	Simpson et al., study 2
	Exploiting error experiences and sources	Productive and vicarious failure, Learning from erroneous examples	

### Contextual factors as an enabler and barrier

This special issue provides further evidence that various contextual factors influence learning from errors. Contextual factors are related to task characteristics, learning context, and the interpersonal dimension of handling errors in social environments (Tulis et al., 2016). Concerning these factors, Darabi et al.'s (2018) meta‐analysis on learning from failures revealed that most experimental research was conducted in grades 6–12 and science/math courses. As suggested by Tulis et al. (2016), the studies of the present special issue addressed issues related to contextual factors from a broader perspective involving primary and secondary school students, parents, teachers, and formal as well as informal contexts.

The interpersonal dimension of the classroom context, in particular the error climate, is considered to play an essential role in learning from errors (e.g. Steuer et al., 2013; Steuer & Dresel, 2015). Prior correlational studies revealed that a more positive error climate led to more affective‐motivational adaptive reactions to errors and then action reactions, which was associated with higher achievement in both German and Italian contexts (Grassinger et al., 2018; Soncini et al., 2022). In this special issue, the longitudinal studies by Dresel et al. (2024) and Steuer et al. (2024) provide insights into how the error climate is associated with the development of students' error‐related reactions and actions and relations with teachers. Dresel et al. (2024) determined that students' affective‐motivational and action‐related reactions differed significantly between classrooms, and the negative development of adaptive reactions over time slowed down in the classrooms with a more positive error climate. Steuer et al. (2024) also highlighted the importance of error climate. They found that primary school students' perceptions of the error climate in class predicted their alienation from teachers 1 year later in the contexts of Switzerland and Luxembourg. In other words, a positive error climate was linked to less alienation from the teachers.

Teachers' mindsets might also be critical in their construction of a context conducive to learning from errors. For instance, Di Battista (2024) examined teachers' gender stereotypes in the context of hypothetical failure moments in educational robotics (ER) courses. Teachers with high levels of gender stereotypes were found to be more hesitant to offer ER to female students. This hesitation pertained to teachers' attribution of errors to female students' low aptitude for ER.

Teachers' mindsets about learning from errors might change with specific interventions. For example, working in an informal learning context, museum educators in Simpson et al.'s (2023) first study named organizational challenges as obstacles to supporting learning from errors in the interviews. Specific challenges included limited time with students, parent expectations, and pressure to produce ‘successful’ products in a paid camp to ensure continuity. In their second study, a professional development program based on video‐based reflections helped museum educators focus more on their own failures and interactions with students by decreasing emphasis on the failures attributed to environment, time, and learner differences. Transforming negative failure experiences into positive and successful ones and providing students with space to make their decisions during failures were also highlighted by the educators in this study to illustrate their shifting mindsets. This finding is in line with prior research targeting teachers' professional development with training programs to enhance their knowledge about specific learner errors, strategies for dealing with errors, and beliefs about the benefits of student errors (Seifried et al., 2015).

Finally, it is essential to note that learning from errors is not confined to only communication between teachers and students; in fact, it also involves parents' conversations with their children at home context, as Peterson et al. (2024) reported in this special issue. Peterson et al. (2024) analysed features of mother–child conversations reminiscing about recent setbacks and revealed low occurrences of mothers' recognition of their children's emotions, discussion of action plans, and collaborative ways of working to avoid future setbacks in these conversations. The results also indicated that children had less fear of failure when their mothers' conversations included clear emotional recognition and commitment statements for collaboratively handling failures. While making an action plan was related to increasing fear of failure, combining this plan with a parental commitment to work together to avoid future setbacks was associated with a decreased fear of mistakes, highlighting the importance of feeling supported when engaging with mistakes.

### Individual factors in Learners' processing of errors

Individual factors can influence reactions to and learning from errors, as indicated in the models by Tulis et al. (2016) and Zhang and Fiorella (2023). In prior work, several motivational beliefs and emotions (e.g. goal orientation, academic self‐concept, anxiety, fear of failure, task value, attitudes toward making errors, and causal attributions to success) have been found to be associated with learners' cognitive, behavioural, affective, or motivational reactions to errors (Klopp et al., 2013; Lauzier & Bilodeau Clarke, 2023; Narciss et al., 2014; Steuer et al., 2013; Tulis & Ainley, 2011). Furthermore, metacognition research has investigated the role of learners' response confidence and revealed the hypercorrection effect (i.e. learners with high response confidence are more likely to correct their errors after feedback; Butterfield & Metcalfe, 2001; Metcalfe, 2017). From the cognitive perspective, prior knowledge has been the focus of interest and was found to be positively correlated with learners' corrections of errors (e.g. Sitzman et al., 2014, 2015).

Several papers in this special issue contributed further findings on the role of individual factors in learners' reactions to and processing of errors. Concerning age and grade, Dresel et al. (2024) determined a significant decrease in students' affective‐motivational and action‐related adaptive reactions to errors in math when they passed from 5th grade to 6th grade.

Three papers provided new findings related to learners' motivational beliefs and emotions. First, Peterson et al. (2024) found lower levels of fear of making mistakes when 8‐year‐old children had higher self‐worth. Second, Sharabi and Roth (2024) examined adolescents' emotional regulation styles as an individual factor in two studies. Both studies revealed positive correlations between integrative emotional regulation and adaptive responses to failures in math (e.g. seeking instrumental and emotional support and learning failure). On the other hand, suppressive emotion regulation was correlated with blaming others and rumination, and emotional dysregulation was associated with defensive coping mechanisms (e.g. denial, blaming others, and rumination). Finally, DeLiema et al. (2024) found differences in students' and instructors' stances and values at failure points during debugging tasks in computer science workshops, which indicated individual differences in the way of interpreting and handling failures. DeLiema et al. (2024) recommended that educators listen to their students' voices when they have diverging approaches to learning from failures and discuss these differences.

Error‐related attitudes were included in the two studies by Tulis and Dresel (2024) as well as Schmid et al.'s study (2024). The first study by Tulis and Dresel (2024) examined if and how error‐related beliefs can be changed through a brief intervention revealing the benefits or harms of errors for further learning. The findings indicate that a brief intervention is not enough to alter error‐related beliefs over a longer period of time. Schmid et al. (2024) found similar results: in their study, students' error orientation remained stable from the beginning to the end of an intervention which informed students about error types and a constructive approach to handling errors in programming tasks.

### Error‐ or failure‐related learning processes

To learn from errors or failures, learners need to process information and regulate their behaviour on several levels (cognitive, metacognitive, motivational, emotional, affective, behavioural, and social). Several theoretical frameworks have synthesized insights on error‐related learning processes and the individual and contextual factors influencing these processes (e.g. Tulis et al., 2016; VanLehn, 1988, 1999; Zhang & Fiorella, 2023). Among the earlier theories, the Cascade theory (VanLehn, 1988, 1999) highlights that task difficulties leading to impasses (i.e. failure experiences where learners cannot attain their goals due to inadequacy of existing knowledge or uncertainty about the appropriate rule) can result in different kind of errors if students use a muddling through strategy instead of the task‐oriented cognitive strategy of trying to understand the reasons for the impasse and figuring out systematically how to overcome the impasse. Reflecting on the origins of also illustrated changes in students these errors (e.g. through self‐explanations or with the help of analogue task examples) and finding ways to repair them is considered crucial for constructing accurate rules and developing strategies that can be transferred to other tasks (VanLehn, 1988, 1999; Ohlsson, 1996a, 1996b). Seeking help from others or receiving and mindfully processing feedback from external sources can also facilitate learning and application of correct rules (Narciss, 2008; VanLehn, 1988, 1999). DeLiema et al. (2024) provide further evidence of these processes by investigating the debugging and learning processes of three middle school students over 1 year and analysing the discourses between students and teachers of a programming course. Five processes during debugging erroneous computer programming codes were identified and titled as: fixing the bug, avoiding recurring bugs, growing debugging skills, engaging with authorities, and calibrating self‐efficacy. DeLiema et al. (2024) also illustrated changes in students' emotions and self‐efficacy during debugging. For example, one student's expressions indicated his negative emotions (e.g. frustration or disappointment) and low self‐efficacy when he could not debug effectively and revealed positive emotions (e.g. pride and joy) after he successfully debugged the broken codes.

Zhang and Fiorella (2023) proposed a model that explains two main stages of learning from one's own errors from a cognitive and a self‐regulated learning perspective. The first stage consists of working on tasks and generating errors, and the second of detecting and correcting errors. During task processing and generating errors, prior knowledge about the content and self‐regulated learning plays a core role. Depending on students' level of prior knowledge, the errors they generate can be semantically related or unrelated to the content. In the second stage, reference information (e.g. feedback, instruction, and rubrics) is crucial for detecting and correcting errors. If students' errors are semantically related to the content, comparing their answers with reference information helps them generate internal feedback and understand these errors. On the other hand, when semantically irrelevant errors are generated, this stage yields low‐quality internal feedback and repair of surface errors, leading to minimal knowledge gains. Metcalfe et al.'s finding (2024) that the time teachers spent with their students on reasoning interactively on errors and their corrections contributed to learning benefits, provides insights into how the second phase can be supported by interactive teaching and feedback strategies.

Extending the models with a focus on cognitive error processing, Tulis et al. (2016) presented a model explaining emotional, motivational, and self‐regulatory processes during learning from errors by considering personal and contextual conditions. According to this model, the starting point of the learning process is detecting errors or receiving feedback, which might trigger learners' direct affective reactions to errors (e.g. frustration and surprise) and prompt them to evaluate the error situation. Depending on how learners attribute the causes of the errors and perceive their personal control level and resources, they can also show indirect affective reactions. Negative emotions and low self‐ and task‐ related motivation due to errors can result in using different emotional and motivational regulation strategies, either effective (i.e. adaptive) or dysfunctional (i.e. maladaptive), to sustain learning motivation and take cognitive, metacognitive, and behavioural actions during the learning process. If effective strategies are implemented, learners might enhance their knowledge, performance, and skills, which also influence their personal conditions (e.g. knowledge and motivation) and subsequent error experiences.

Tulis et al.'s (2016) model was used as a theoretical framework by several papers of this special issue (Dresel et al., 2024; Soncini et al. (2024); Tulis & Dresel, 2024). The longitudinal study by Dresel et al. (2024) investigated how perceived error climate influenced the development of students' reactions to errors in math classes from the beginning of 5th grade until the end of 6th grade. Findings revealed that, on average, there was a negative development of students' affective‐motivational and action‐related reactions to errors. Yet, the extent of this negative development was less in classrooms with a more positive perceived error climate. In their experimental study, Soncini et al. (2024) investigated how supportive vs. discouraging feedback affected students' affective‐motivational reactions to errors and contributed empirical evidence on the role of feedback in students' reactions to errors. The two experimental studies by Tulis and Dresel (2024) aimed at altering error beliefs with (a) general prompts (error‐belief related—study 1), and (b) direct prompts (concretely related to affective‐motivational reactions and/or to actions, study 2) and investigated if and how the belief changes influenced students' reactions to errors. The findings of the second study revealed the benefits of providing students with concrete prompts concerning adaptive reactions to errors or failures.

Inspired by the Self‐Determination Theory (Ryan & Deci, 2017), Sharabi and Roth (2024) examined correlations among students' emotion regulation styles (integrative vs. suppressive) and their coping practices (adaptive vs. maladaptive = defensive) in response to academic failure (study 1), as well as self‐reported outcomes from learning from failures (study 2). In both studies, an integrative emotion regulation style was associated with adaptive coping practices (positive reinterpretation of errors in terms of growth opportunities, instrumental and emotional help‐seeking). Furthermore, the second study revealed that the effect of integral emotion regulation on learning from failures and on cognitive engagement with math was mediated by adaptive coping practices.

### Instructional strategies to support learning from errors

Considering the complex and dynamic learning process from errors, supporting students' cognition, metacognition, motivation, and emotions to facilitate learning from errors becomes critical. To this end, different instructional strategies are suggested in the literature (e.g. feedback, Metcalfe, 2017; Mera et al., 2022; prompts, Siegler, 2002; and productive failure; Kapur, 2010, 2014a, 2014b). In this regard, the effects of feedback, prompts, and belief inductions were investigated in the papers of this special issue (e.g. Metcalfe et al., 2024; Schmid et al., 2024; Soncini et al., 2024; Tulis & Dresel, 2024).

Feedback (strategies) can be designed in manifold ways and differ in terms of functional (e.g. cognitive, metacognitive, and motivational), content‐related (e.g. evaluative and elaborated components), and formal characteristics (e.g. modality, frequency and timing) (Narciss, 2008, 2017). Therefore, it is essential to investigate under what conditions and which kind of feedback strategy can best unfold its benefits. Metcalfe et al. (2024) addressed this issue by comparing explicit instruction with instruction based on learning from errors and found higher teaching efficiency in error‐based instruction. More in‐depth analysis of error‐based instruction also revealed the benefits of a teaching style in which teachers use interactive feedback strategies that on the one side prompt students to reflect on the reasons for errors and on how to correct them, and on the other side adapt their external feedback to the needs of the students enhanced 8th graders math‐learning. This finding confirms and extends prior findings on the role of elaborated feedback in learning from errors (e.g. Mera et al., 2022; Metcalfe, 2017). Altogether the present findings provide further empirical evidence for a core implication of the Interactive Tutoring Feedback model, namely that feedback strategies should be designed in an interactive way to empower students' learning (from errors) (Narciss, 2008, 2017).

In their experimental study, Soncini et al. (2024) manipulated the presentation of feedback provided to errors in a computer‐based training to examine the impacts of providing the same feedback content in a supportive or discouraging form on middle school students' reactions toward errors in online homework. They found that the students receiving supportive feedback for their errors through smileys and motivating sentences had more conducive affective‐motivational reactions toward their errors than those receiving discouraging error feedback. There was also an indirect effect of supportive feedback on action reactions toward errors via affective‐motivational reactions toward errors. This finding confirms the theoretical consideration that it is important to carefully design not only the feedback content but also the form and mode it is provided (Narciss, 2008, 2013, 2017).

Providing instructional prompts is also one strategy that can trigger students' cognitive, metacognitive, motivational, and collaborative processes during the regulation of learning (Bannert, 2009). First, in Tulis and Dresel's first study (2024), the researchers designed booklets with learning tips about either the benefits or drawbacks of errors for the intervention groups before a computer‐based 50‐minute learning session. These interventions can be called induction of positive‐ or negative error‐related beliefs, respectively. The group inducted to positive beliefs had higher beliefs about learning from errors than those inducted to negative beliefs directly after the manipulation. However, the impact of positive induction on beliefs decreased after 50 min. In contrast, the negative induction to learning from errors had a more stable effect by causing less error‐adaptive responses (i.e. action‐related reactions, metacognitive control, and persistence). Furthermore, there were no significant differences in knowledge gains between intervention and control groups.

In their second study, Tulis and Dresel (2024) used prompts to induce adaptive affective‐motivational reactions and adaptive action‐related reactions after error feedback. Although there was no significant difference between the intervention groups and the control group without prompts in regard to knowledge gains, Tulis and Dresel (2024) revealed that the group with affective‐motivational prompts had higher persistence than the control group. Moreover, action‐related prompts enhanced undergraduate students' metacognitive control level, intentions to take adaptive action in response to errors, and effort investment.

Schmid et al. (2024) designed a one‐day visual programming workshop about developing smart textiles for secondary school students. Different from the control group attending the regular workshop, the intervention group was informed about error types and a constructive approach to handling errors after each programming task. The results revealed that students' enjoyment increased, and anxiety and boredom decreased in both groups over time; however, the intervention had no significant effect on the change of these emotional states during the tasks. Moreover, students' error learning orientation remained almost stable from the beginning to the end of the intervention.

As mentioned above, these findings (Tulis & Dresel, 2024; Schmid et al. 2024) indicate that short interventions that aim to change students' error‐learning orientation or beliefs about errors are not strong or comprehensive enough to contribute to long‐lasting changes of error‐related beliefs. The prompts that target concrete tasks and error‐related behaviour, like in the second study by Tulis and Dresel (2024), seem to be more promising. This finding is in line with prior research using cognitive prompts supporting information processing (e.g. by asking students to generate internal feedback in terms of self‐explaining why the answer is correct or incorrect; Narciss et al., 2022).

## CONCEPTUAL ISSUES, CHALLENGES, AND IMPLICATIONS

Despite the notable strength of this set of papers, several conceptual issues and challenges that are worth to be considered in further research became apparent. In the following, we will address the ones we consider most relevant and suggest some new directions for further inquiry.

### Error and failure experiences—How similar or different are they?

The first conceptual issue is indicated by the title of the special issue: ‘Learning from errors and failure in educational contexts’ as well as across the titles of the special issue papers (some use the error term, others the failure term). The terms error and failure have different meanings (seee.g. Simpson et al., 2020). An error is defined as a deviation from a standard (Reason, 1990). Defined in such a generic way, the term *error* refers to all cases in which a planned series of mental or physical activities fail to attain the intended outcome (Reason, 1990). Reason (1990) distinguishes three categories of errors: slips, lapses, and mistakes. Slips and lapses refer to observable or covert errors that emerge from failures in storing or executing the action plan, even if the plan was sufficient to achieve its objective (Norman, 1981; Reason, 1990). For instance, skipping a step due to slips in attention and memory lapses can cause unintended actions. Mistakes arise from deficiencies or failures in the processes of selecting an objective or determining the means to accomplish it. For example, lacking enough declarative, conceptual, and procedural knowledge, having misconceptions, and applying a plan inappropriately for the situation can result in knowledge‐based and rule‐based mistakes (Reason, 1990). These mistakes can also be related to learners' having insufficient negative knowledge (i.e. knowledge about inaccurate definitions, operations, approaches to problem‐solving, and associations that can complement positive or accurate knowledge; Oser & Spychiger, 2005).

Whether errors are experienced and interpreted as a failure (i.e. a negative, shaming experience) depends on numerous factors, as revealed by the systematic review by Simpson et al. (2020). Failure experiences can originate not only from making errors but also from goal‐related setbacks, from encountering obstacles or impasses in problem‐solving, from evaluative feedback, or just through the comparison of one's own performance with those of others. The terms error and failure should, therefore, not be used synonymously to avoid the risk of a jangle fallacy (i.e. using different terms to refer to the same phenomenon). Instead, research on learning from errors needs to address questions such as, ‘by whom, when and/or why is an error considered as a failure’, ‘what kind of errors should be differentiated?’, ‘what kind of failure experiences should be differentiated?’, ‘what kind of errors are considered as a failure?’, in order to investigate under what conditions errors are appraised and used as learning opportunities vs. experienced as (shaming) failure events. The findings of the qualitative studies of this special issue (DeLiema et al., 2024; Simpson et al., 2023) shed some light on the first question and indicate that finding responses to these questions can depend on various factors, including (a) the educational domain or topic, (b) the learning tasks, (c) individual learner and teacher characteristics, as well as (d) contextual factors.

### Error‐related attitudes

Furthermore, conceptual challenges arise from the labelling of some of the core individual factors, which were investigated by various studies. For example, to address and assess individual differences in how errors or failures are perceived and appraised, the following constructs and their related instruments have been used: error learning orientation vs. error avoidance orientation (Schmid et al., 2024); failure mindsets (failure‐as‐enhancing vs. failure‐as‐deficiency mindsets (Simpson et al., 2023); error‐related beliefs (Tulis & Dresel, 2024); emotion regulation styles (integrative vs. suppressive; Sharabi & Roth, 2024). Even though the authors within this special issue refer to the respective articles in this special issue, further research is challenged as it is still unclear what the common and distinct features of these constructs and instruments are and to what extent they could be used interchangeably or should be used for specific contexts (e.g. within cross‐sectional or longitudinal studies), or from a specific theoretical perspective. For further research, it would be valuable to systematically revisit what kind of individual differences constructs have been used in research on learning from errors and/or failures and examine if and how they share distinct or common conceptualizations of their (a) stability vs. variability or malleability, (b) specificity (e.g. general, domain, task), (c) dimensionality (how many dimensions), and (d) target person(s) (e.g. learner, teacher, educator, parent; individual vs. collective).

### Error‐related affective‐motivational reactions and actions

Moreover, using different theoretical perspectives, the studies have investigated diverse reactions and actions from diverse stakeholders in response to their own or others' errors and failures. For example, based on Tulis et al.'s model concerning students' reactions and actions to errors (Tulis et al., 2016), in several studies the focus was on *affective‐motivational* and *adaptive action reactions* and how they are influenced by (a) manipulating error beliefs (Tulis & Dresel, study 1) or (b) by prompts inducing either adaptive affective‐motivational reactions or adaptive action related reactions or both types of reactions (Tulis & Dresel, study 2), or (c) encouraging and discouraging error feedback (Soncini et al., 2024), or (d) in the long term by the perceived error climate (Dresel et al.'s longitudinal study). Sharabi and Roth (2024) took a Self‐Determination Theory perspective and investigated the associations among students' emotional regulation styles, their problem‐focused vs. defensive strategies to cope with academic failures, and their learning from failures. In the study by Schmid et al. (2024) that is based on Pekrun's Cognitive Value Theory of Achievement Emotions (Pekrun, 2006), the focus is on the interaction among *error learning orientation* and selected state *achievement emotions* (joy, anxiety, and boredom). Peterson et al. (2024) investigated features of mother–child conversations (e.g. acknowledgment of children's emotions by parents, discussion of action plans, and types of resources) about recent disappointments or setbacks and their associations to children's *fear of failure* based on Covington's Needs Achievement Model (e.g. Covington, 2009) and Ajzen's (1991) Theory of Planned Behaviour. In all these studies, reactions and/or actions to errors are classified into more or less adaptive types based on the frameworks used. Yet, the study by Sharabi and Roth (2024) indicates that, concerning the affective/emotional reactions to errors, a classification in adaptive vs. maladaptive seems to be premature because the impact of the emotional reactions can be mediated by error‐related coping, emotion regulation, or self‐regulated learning strategies.

Furthermore, most of the studies have a selective focus on affective‐motivational reactions. For example, in the studies by Tulis and Dresel, 2024; Soncini et al., 2024 and Dresel et al. (2024), the scale of the self‐report instrument addressing affective‐motivational reactions to errors includes mainly items concerning the effect of errors on experiencing joy in math classes (e.g. ‘if I say something incorrect, the math is still just as much fun for me’). Schmid et al. (2024) focused on the interaction among error learning orientation and the selected state achievement emotions (joy, anxiety, and boredom). However, besides joy, anxiety, and boredom, the epistemic emotions surprise, curiosity, and confusion, as well as the achievement emotions pride and shame, have been found to be prevalent during complex learning and associated with exploratory learning behaviour (e.g. Liu et al., 2022; Vogl et al., 2020; Zhang et al., 2021). Thus, an important issue for further research would be to conceptualize a more differentiated view on potential affective/emotional, and/or motivational reactions to errors and/or failures, taking into consideration and maybe integrating frameworks on achievement and epistemic emotions (e.g. Pekrun et al., 2023; Vogl et al., 2020) and also the self‐conscious affect framework developed by Tagney (1990) and investigated in further studies (e.g. Luyten et al., 2002; Watson et al., 2016, 2017).

Most of the studies also take a rather selective view of the conditions and options for actions following errors. For example, the scale used to gather data on students' actions after errors emphasizes mainly maintaining or increasing effort to strive for improvement (e.g. ‘if I make a mistake, I try deliberately, to improve myself’). Yet, the qualitative study by DeLiema et al. (2024) provides data indicating that the range of actions after errors can be investigated in a task‐specific way, and there might be differing views on the roles of the actions from different stakeholders. Hence, classifying the diverse actions after errors or failures into adaptive or maladaptive might be too simplistic. When and how problem‐focused actions such as maintaining or even increasing effort, maintaining goal‐pursuit, or help‐seeking vs. self‐worth protective actions such as emotion regulation or attention shifting are conducive for learning depends not only on the individual factors examined in the present studies (e.g. error beliefs, error orientation; emotion regulation styles; global self‐worth), but may also depend on (a) students' cognitive, metacognitive and motivational pre‐requisites (e.g. knowledge, learning strategies, confidence, task value, self‐efficacy), (b) on task characteristics (e.g. task complexity and/or difficulty), and (c) contextual factors (e.g. time constraints, access to and availability of resources). Accordingly, future studies should take into consideration a broader range of potential emotions after errors, as well as a broader range of action options in order to investigate the issues of (a) under what individual and situational conditions and (b) how achievement, epistemic, and/or self‐conscious emotions after errors and/or academic failures benefit or harm learning.

## METHODOLOGICAL APPROACHES, CHALLENGES AND IMPLICATIONS

The 11 papers of this special issue use a range of research methods (see Table 2 for an overview). In the following sections, we will first provide a brief overview of the approaches, then discuss selected challenges and implications for further research related to these approaches.

**TABLE 2:** An overview of methodological approaches of the papers in the special issue.

Authors/study	Design	Domain/task	Participants/stakeholders	Context/setting
DeLiema et al. (2024)	Case study with 3 students	Programming	Middle school students and their teachers	Workshop—Computer Science
Simpson et al. (2023)	Qualitative—Interviews (study 1)—Video analysis (study 2)	Science, Professional development	Science educators	Middle school Science museum – maker‐space
Dresel et al. (2024)	Correlational longitudinal	Math	5th–6th grade students	School
Steuer et al. (2024)	Correlational longitudinal	Across domains	5th–6th grade students	School
Sharabi and Roth (2024)	Correlational	Math	8th–12th grade students	School
Peterson et al. (2024)	Correlational	N. A.	Mother–8‐year‐old child dyads	Home
Metcalfe et al. (2024)	Longitudinal quasi mixed model within subjects design	Math	Teachers and their 8th grade students	School
DiBattista et al.	Experimental	Educational robotics	Teachers	School–[vignettes–gender]
Schmid et al. (2024)	Experimental	Programming	7th–9th grade students	Workshop–visual programming
Soncini et al. (2024)	Experimental	Civil education	6th–8th grade students	Online homework
Tulis and Dresel (2024)	Experimental (2 studies)	Research methods—Statistics	Undergraduate teacher education students	University

### From qualitative case studies to experimental designs

Both qualitative and quantitative (i.e. correlational and experimental) as well as mixed study design were employed in the research in this issue. In qualitative studies, DeLiema et al. (2024) investigated the debugging and learning process of three middle school students by analysing discourses between students and teachers, and Simpson et al. (2023) explored educators' beliefs about learning from failures and practices before and after attending a professional development program. Correlational studies sought the relationship between error climate and students' reactions to errors (Dresel et al., 2024) and alienation from teachers (Steuer et al., 2024), emotional regulation style and coping with academic failure (Sharabi & Roth, 2024), teaching style and student learning (Metcalfe et al., 2024), and mother–child conversations and children's fear of failure (Peterson et al., 2024). Experimental studies (Di Battista, 2024; Metcalfe et al., 2024; Schmid et al. (2024); Soncini et al., 2024; Tulis & Dresel, 2024) analysed the effect of instructional strategies (e.g. supportive error feedback and prompts about how to approach errors) and the gender of students making errors on cognitive, metacognitive, motivational, and emotional outcomes.

### From programming to civil education

In contrast to prior research that has mostly focused on math and science (Darabi et al., 2018), the studies in this special issue addressed different subject domains where errors occur. Four studies were conducted in the domain of computer programming (DeLiema et al., 2024; Di Battista, 2024; Schmid et al., 2024; Simpson et al., 2023), three in math (Dresel et al., 2024; Metcalfe et al., 2024; Sharabi & Roth, 2024), and one in research methods (Tulis & Dresel, 2024) and civil education (Soncini et al., 2024).

### From students to parents

Different stakeholders accountable for learning from errors were considered in this special issue. Seven were conducted with K‐12 students from primary and secondary schools (DeLiema et al., 2024; Dresel et al., 2024; Metcalfe et al., 2024; Sharabi & Roth, 2024; Schmid et al., 2024; Soncini et al., 2024; Steuer et al., 2024). Undergraduate students (Tulis & Dresel, 2024), teachers (Di Battista, 2024; Simpson et al., 2023), and mothers (Peterson et al., 2024) were involved in the other studies.

### From schools to informal learning settings

The learning settings for students also varied in this special use. They consisted of school courses, including after‐school tutorial programs and online homework, and some informal settings, such as programming workshops and science museums (DeLiema et al., 2024; Schmid et al., 2024; Simpson et al., 2023). Overall, this special issue also indicates that learning from errors is not confined to only formal learning contexts.

### Challenges and implications

The summary of the research approaches illustrates that research on learning from errors or failures can be conducted in manifold ways. The diversity of methodological approaches also reveals several challenges researchers face while investigating learning from errors. Some of these challenges are linked to the conceptual challenges discussed above. For example, a more differentiated and comprehensive conceptualization of affective/emotional, and/or motivational reactions and actions to errors would also require revisiting the existing measurement instruments and strategies for assessing these reactions and actions in order to inform future researchers on the shared and distinct characteristics of existent instruments, and by doing so extend the chance that the database gathered with these instruments is increased, while at the same reducing the risk of redundant ad hoc developments and usages of scales (as for example in Sharabi and Roth, 2024). Furthermore, future research would benefit if, based on a more differentiated conceptualization of error‐related reactions and actions, existent instruments targeting the same reactions or actions would be integrated or modified, or new scales would be developed, and the resulting instruments and strategies validated.

Other challenges result from the complex interplay of more or less generic vs. specific individual and more or less distal or proximal contextual factors influencing how errors are perceived, processed, and dealt with by the different stakeholders (i.e. learners, teachers, peers, parents, and educators). For example, several studies examined the issue of whether and how error‐related individual rather generic dispositions such as error‐related beliefs or error learning orientation can be changed through interventions (e.g. emphasizing the benefits of errors, Tulis & Dresel, study 1; prompting constructive strategies to process the errors, Schmid et al., 2024). Other studies focused on the issue of whether and how specific error‐related individual reactions and behaviour can be influenced through interventions (e.g. prompting adaptive affective‐motivational or action‐related reactions, Tulis and Dresel, study 2; supportive or discouraging feedback, Soncini et al. (2024). The findings indicate that the generic individual dispositions are rather stable, while more specific error‐related individual reactions and behaviour are malleable. Thus, future studies should investigate what kinds of interventions have beneficial effects on malleable error‐related reactions and actions rather than on factors on the disposition level and how these changes are associated with error‐related learning or knowledge gains. The experimental studies by Soncini et al. (2024) and Tulis and Dresel (2024) illustrate well‐founded approaches that can be used to profitably orient future research in this direction.

Concerning the effects on reactions and actions following errors, it should be noted, however, that in most of the studies, they were assessed with self‐report instruments. Behavioural process data were only captured in the qualitative studies and in the longitudinal mixed‐method study by Metcalfe et al. (2024). Further studies that examine correlates among individual dispositions and reactions and actions following errors and/or the effects of instructional support or interventions should also collect concrete error‐related behavioural process data. For example, the kind of behaviours that could be of particular interest in diverse learning contexts on the learner side might be derived from research on learning from erroneous examples (e.g. error detection, error reflection and explanation, error correction; comparison with correct examples) (e.g. Adams et al., 2014; Barbieri & Booth, 2020; Yang et al., 2016).

Besides students' reactions and actions to errors and failures, diverse associations between enabling or harmful reactions and actions from teachers and parents and short as well as long‐term effects or consequences for students have been investigated. For example, Metcalfe et al. (2024) investigated in an ecological setting over 2 years under what teaching styles (interactive vs. directive) different experimentally induced instructional strategies (explicit instruction vs. error‐directed feedback strategies) are associated with performance in the high‐stakes New York State Algebra 1 Regents examination. The longitudinal mixed‐method study by Metcalfe et al. (2024) reveals that interactive error‐directed feedback strategies that engage students in understanding their errors and correcting them are more conducive than directive strategies focusing on getting quickly to the correct solution. Future research on the conducive behaviour of teachers, parents, and educators could benefit considerably from using the findings from the Metcalfe study and from studies on formative interactive feedback strategies (e.g. Laudel & Narciss, 2023; Narciss et al., 2022) as a source of inspiration for the selection of error‐related reactions and overt behaviours. These more proximally error‐related behaviours could, at the same time, serve as a starting point for future design‐based research aiming at designing and evaluating further intervention strategies, materials, and tools providing scaffolds, prompts, feedback, scripts, or combinations of them to support all stakeholders to use errors and/or failures as learning opportunities.

## SUGGESTIONS TO ENHANCE EDUCATIONAL PRACTICES

One essential and preliminary condition for learning from errors might be a positive error climate in both formal and informal educational settings. As indicated in this special issue by several studies, a positive climate can help students show more adaptive reactions to errors, maintain positive emotions and attitudes toward teachers, and enhance subject knowledge (Dresel et al., 2024; Metcalfe et al., 2024; Steuer et al., 2024). A positive error climate in classrooms can be created with (1) teachers who do not avoid students' mistakes, do not penalize students for their errors, and provide support and positive reactions; (2) classmates who do not give negative reactions to errors and support their peers making an error; and (3) social processes that allow analysis and discussion of errors to enhance learning (Steuer et al., 2013).

For informal education at home, parents' and siblings' approaches to children's errors also need to be considered. These agents' supportive interactions can have a complementing or even more substantial impact on children's learning from errors. For example, Raftery‐Helmer and Grolnick (2018) found that compared to teachers' autonomy support, parents' autonomy support was significantly and positively correlated with children's thinking of failure as a challenge and negatively correlated with children's thinking of failure as a threat. Parents' involvement behaviours in children's education were also found to be related to children's use of mastery coping strategies in academic failures (e.g. help‐seeking and problem‐solving). However, it might not be very likely for parents to respond to the needs of their children in productive ways (e.g. recognizing their children's emotions and discussing action plans for error correction) at failure points, as Peterson et al. (2024) revealed by analysing mother–child conversations in this special issue. Hence, it is also of great importance for parents to learn and apply supportive behaviours to help their children learn from errors.

This section explains potential strategies to foster a positive error climate and learning from errors considering the roles of different agents (i.e. teachers, parents, and peers) and the results of the studies in this special issue, as well as related research addressing the learning from errors. They include (1) training programs to develop error competence, (2) exploiting error experiences and various sources of errors, (3) instructional tools supporting learners' processing of errors, and (4) dialogues about errors. These strategies can be used individually or combined to present a more comprehensive support structure.

### Training programs to develop error competence

To construct an error climate conducive to learners' processing of errors, teachers, peers, and parents should have the competencies necessary to facilitate learning from errors. According to Wuttke and Seifried (2017) ‘professional error competence’ involves three components: (1) knowledge of students' domain‐specific errors and their potential causes, (2) strategies for effective handling of errors (e.g. identifying actual causes of errors and giving quality feedback), and (3) beliefs about the benefits of handling errors in classes. However, teachers might not have strong professional error competence, as revealed in this special issue. Their core beliefs concerning gender stereotypes (Di Battista, 2024), disregard of students' perspectives at failure moments (DeLiema et al., 2024), and use of lecturing rather than more interactive instruction for error analysis and correction (Metcalfe et al., 2024) can lessen the learning opportunities from errors. Therefore, professional development programs for teachers can be prepared to enhance their error competence. These programs can be grounded on the frameworks of dialogic teaching (Alexander, 2008), feedback literacy (Carless & Winstone, 2023), and learning from errors (e.g. Oser & Spychiger, 2005; VanLehn, 1988, 1999) to empower teachers' pedagogical content knowledge about errors. Furthermore, as Simpson et al. (2023) suggested, teachers' reflections on their videotaped lessons with their colleagues might help them analyse and shift their beliefs and practices in these programs.

Students in classrooms can also be trained to promote their task and domain knowledge and affective and strategic competence in dealing with their peers' errors, especially in peer assessment activities (Chen, 2016). More particularly, this training can aim to inform students about assessment criteria with rubrics and examples, the educational value of peer assessment, and how to give feedback on peers' errors and respond to peer feedback in a supportive and negotiable way considering the features of student feedback literacy (Carless & Boud, 2018). Prior research showed the positive influence of such training on mitigating students' concerns and anxiety about providing and receiving peer feedback, generating higher quality peer feedback, and increasing perceived learning and performance (Alemdag & Yildirim, 2022a, 2022b; Alqassab et al., 2018; Li, 2017; Li et al., 2020).

Finally, as indicated by the findings of Petersen et al. (this issue) it is necessary to develop training programs targeting parents' knowledge, skills, and beliefs about their children's errors. To illustrate, parents' beliefs that failures enhance learning and growth might be promoted in these programs because they can affect both their reactions to children's failures and children's growth mindset (Haimovitz & Dweck, 2016). Another aim of the training programs might be to develop parents' skills in meeting children's needs of autonomy, competence, and relatedness according to the self‐determination theory, as they were found to be related to children's beliefs about failures and coping strategies (e.g. Ng et al., 2004; Meyer et al., 2019; Raftery‐Helmer & Grolnick, 2018). Specifically, when children encounter failures, parents can be guided to provide them with support in types of (1) autonomy by encouraging their children's independent problem‐solving and participation in decision‐making considering their developmental characteristics (it is important to note that parent–child collaboration might be necessary at lower child ages (e.g. 8‐years old) to prevent future failures as in Peterson et al.'s study), (2) structure by presenting clear and consistent expectations, guidelines, and feedback, and (3) involvement by showing interest and participation in children's life (Grolnick & Ryan, 1989).

### Exploiting error experiences and various sources of errors

Error experiences can be exploited in manifold ways taking into consideration the sources of errors: (1) individual, (2) group/team, and (3) others.

First, individual errors are the errors made by individuals unintentionally or deliberately. Unintentional or ‘naturalistic’ errors occur even if learners aim to attain the correct response, but work, for example, on tasks they lack the necessary knowledge for (Wong & Lim, 2022a). This type of error seems to have occurred, for example, in the programming settings used by DeLiema et al. (2024) and Schmid et al. (2024). Inducing such unintentional errors explicitly by placing a challenging problem‐solving task before providing the instruction on how to solve it has been examined in productive failure studies as an instructional strategy supporting learning from errors (here referred to as failures; Kapur, 2010, 2014a, 2014b). Such a sequencing of problem‐solving and instruction can help students better process new concepts, become aware of knowledge gaps and inconsistencies, feel agency in learning, and activate prior knowledge (Kapur, 2014b). Accordingly, Kapur (2014a, 2014b) found that the students in the productive failure conditions had higher posttest scores than those who received instruction first and learned from peers' unsuccessful problem‐solving attempts (i.e. vicarious failure).

The term ‘deliberate error’ refers to errors that emerge when the instructional setting requires students to make errors even though they know the correct answer. Deliberately making a mistake and then correcting it, even when one knows the correct answer, has been found to be more beneficial for concept learning than highlighting concepts, creating concept maps, paraphrasing concepts, or generating real‐world examples for the concept (Wong, 2023; Wong & Lim, 2022a, 2022b).

Second, in particular in formal educational contexts, many errors occur while students learn together in groups. Errors made by a group of people are also referred to as team errors. These errors originate from individual or shared errors in the team (Sasou & Reason, 1999). Even if the error is generated by an individual alone in the team, team members' cooperation in detecting errors, drawing attention to errors, and correcting them is necessary to recover from errors (Sasou & Reason, 1999). Accordingly, Tjosvold et al. (2004) found that team members' cooperative goal setting and use of a problem‐solving approach involving open discussions of errors and their origins, as well as potential corrective and preventive actions, were significantly associated with their learning from mistakes.

Finally, others' errors presented via erroneous examples, modelling examples, or peer works in peer assessment can be one source for learning from errors vicariously. Erroneous examples include incorrect solution step(s) to a problem in writing and usually prompt learners to detect and correct errors (Adams et al., 2014; McLaren et al., 2015). Similarly, modelling examples present incorrect steps for solving the problem and show this either as live modelling or via video (van Gog et al., 2019). Determining errors in peer works and providing peer feedback also allows students to learn from others' errors and generate internal feedback for their performance (Narciss, 2017; Nicol & McCallum, 2022). Analysing other's errors in these ways can mitigate negative emotions (e.g. shame and anger) students might feel while learning from their errors and motivate deep processing of errors (Khasawneh et al., 2022; Yang et al., 2016). However, as Wong (2023) found, compared to analysing other's errors, generating one's own errors might be more likely to activate learners' internal mental structures and facilitate encoding and integration of the following reference information (e.g. error feedback) (Mera et al., 2022; Metcalfe, 2017; Zheng & Fiorella, 2023). Accordingly, there are conflicting findings regarding the impact of erroneous examples on learning compared to correct examples and problem‐solving (Barbieri et al., 2023; Beege et al., 2021).

### Instructional tools supporting Learners' processing of errors

This special issue presents empirical evidence concerning how individual factors (e.g. grade, self‐worth, and emotion regulation styles) influence learning from errors (Dresel et al., 2024; Peterson et al., 2024; Sharabi & Roth, 2024). Therefore, to optimize learning opportunities from errors for all students, educators and instructional designers need to analyse learner characteristics, adapt the instruction, and provide instructional tools supporting error processing and regulation of learning. These tools include instructional materials, prompts, technological environments, and assessment instruments (Allal, 2016) that can indirectly teach learning strategies. Digital learning environments giving encouraging feedback (Soncini et al., 2024) and prompts to induce affective‐motivational and action‐related adaptive reactions after errors (Tulis & Dresel 2024) have been suggested in this special issue. Other tools that can facilitate learning from errors involve rubrics and metacognitive prompts.

Rubrics that list assessment criteria and performance level descriptions have been found effective in enhancing academic performance, self‐regulated learning, and self‐efficacy (Panadero et al., 2023). These tools can help students detect and correct their own and other's errors, especially in complex performance tasks. For instance, Safadi (2022) found that the students using a rubric to analyse erroneous examples about geometric optics with self‐explanation prompts obtained higher learning gains and were more likely to correct their naïve ideas than those using only worked examples. The mere presence of rubrics, however, might not be enough to enhance learning from errors, and some metacognitive activities via modelling, prompts, and instruction should be accompanied by this tool to guide learners (Panadero & Jonsson, 2013).

Metacognitive prompts that ask students to plan, monitor, control, and reflect on their learning processes (Bannert, 2009) can also support the processing of errors and learning from them. To illustrate, Theobald et al. (2024) used reflection prompts that asked children to relate their answers to what they have already learned in prediction tasks. For incorrectly predicted tasks, the authors found that the group with reflection prompts displayed better conflict monitoring measured with error‐related response times and pupil dilation and revised their incorrect beliefs faster. In the peer assessment context, Alemdag and Yildirim (2022b) revealed that goal setting and planning scaffold high school students used for their writing process helped them detect errors in peers' writings.

Another critical issue in supporting learning from errors with these tools might be how long they should be provided. Brief interventions with instructional tools seem ineffective in obtaining an immediate and lasting effect on students' error‐related beliefs and learning, as the studies in this issue (Schmid et al., 2024; Tulis & Dresel, 2024) revealed. Prolonged interventions can be designed to allow learners to internalize cognitive, metacognitive, motivational, and emotional adaptive strategies while handling errors. It can also help students better observe the utility value of learning from errors, which is required in belief change (Sharot et al., 2023). Lastly, it is crucial for more knowledgeable agents to monitor learners' progress continuously and to remove the support when learners no longer need it to process errors adaptively (Puntambekar, 2022).

### Dialogues about errors

Learners' dialogues with their teachers, peers, and parents about their errors can also contribute to a positive error climate and learning from errors. These dialogues can be regarded as soft scaffolds that can offer dynamic and situational support (Ge & Land, 2004).

Concerning teacher‐student dialogues, in this special issue, Metcalfe et al. (2024) emphasized teachers' interactive discussion with their students on the nature of their errors (i.e. their reasons and error detection and prevention strategies) after common student errors in classrooms were identified. However, teachers' analysis of errors in large classrooms might be challenging. Tools described in the previous part or digital learning environments that automatically record students' errors and report common errors to teachers via a learning analytics dashboard can both help students regulate learning and facilitate teachers' in‐class discussions about errors (Allal, 2016). Another specific strategy to promote in‐class dialogues about errors might be using erroneous examples. For instance, in the study by Safadi and Yerushalmi (2014), after the students detected errors in incorrect examples, the teacher asked them to explain the errors in a classroom discussion, which resulted in the elicitation of different naïve ideas and productive dialogues among peers.

Dialogic peer assessment that involves discussion between peer feedback providers and receivers can enhance learning from errors as well. More particularly, these dialogues allow feedback receivers to get more information about their errors and reasons and suggestions to correct them from their peers (Wood, 2021; Zhu & Carless, 2018; Zhu & To, 2022). Furthermore, feedback receivers might acquire back‐feedback about the accuracy of their error detection and correction (Alemdag & Yildirim, 2022a, 2022b; Zhu & To, 2022). Such dialogic peer assessment administered via online tools has been found effective in improving students' task performance, feedback quality, metacognitive awareness, and self‐efficacy (Kim & Ryu, 2013; Zheng et al., 2018).

Finally, parents' supportive dialogues are an important factor that affects learners' reactions to errors. According to Peterson et al.'s findings in this special issue, conversations with clear emotional recognition and action plans based on parent–child collaborative work can decrease the fear of failure. In addition, prior research (e.g. Ng et al., 2004; Meyer et al., 2019; Raftery‐Helmer & Grolnick, 2018) suggests dialogues that meet children's autonomy needs, do not involve controlling or punishing statements, and provide guidelines and feedback. Training programs and instructional tools (e.g. rubrics) explained in previous parts can guide parents in leading such dialogues with their children.

**FIGURE 1:**
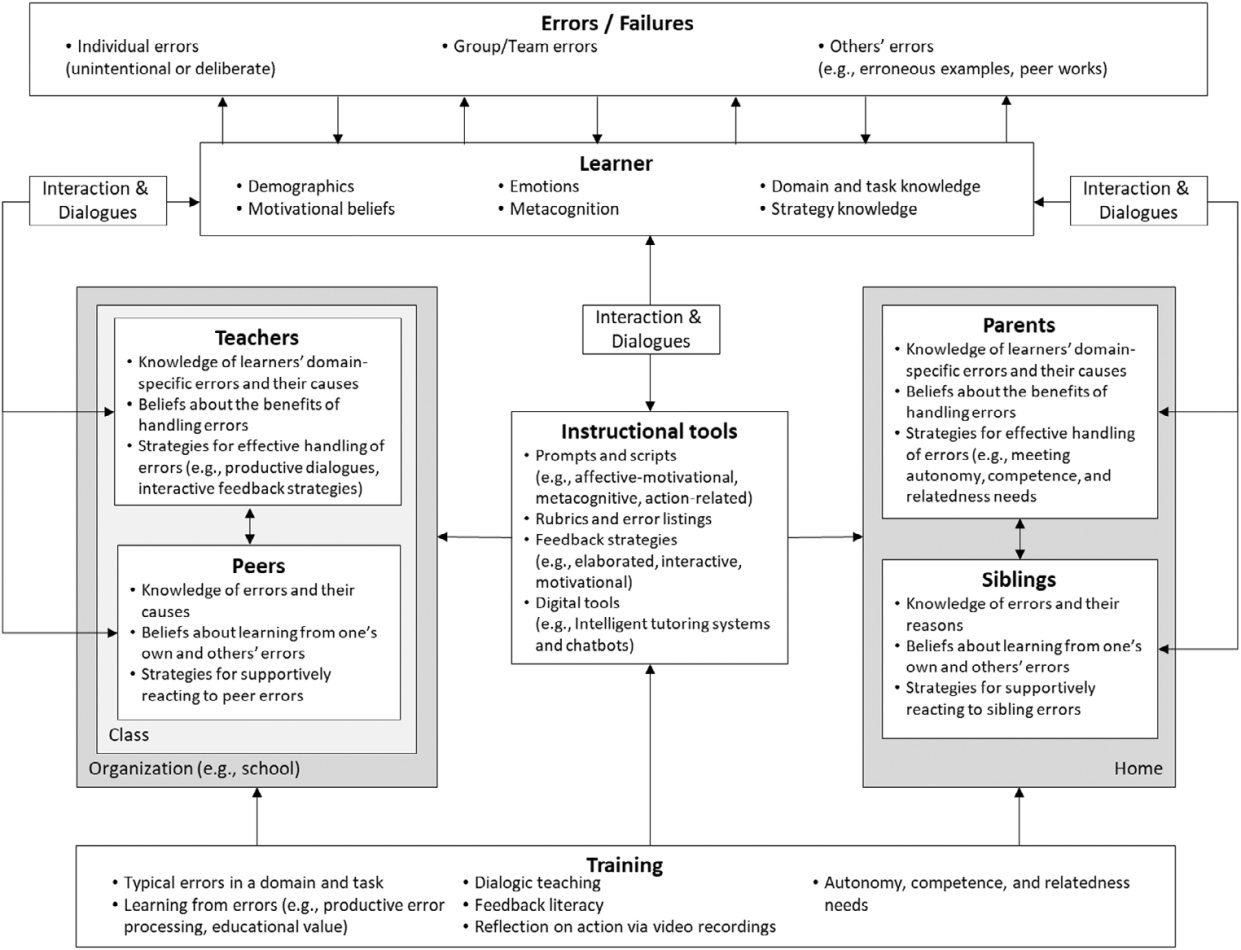
Overview of components of a supportive error climate in educational contexts.

Overall, this paper provides several suggestions for promoting educational practices by taking into account different components of error climate. Figure 1 provides an overview of these components. Dialogues between learners and instructional tools are also indicated in Figure 1. Considering the recent rapid advancements in the use of artificial intelligence in education, interactive digital tools such as intelligent tutoring systems and chatbots trained with data about common student errors, their reasons, and how to respond to them can be a dialogue partner for learners as well (Shih et al., 2023; Siemer & Angelides, 1998).

## CONCLUSION

The collaborative effort of these research groups in presenting their studies together in this special issue has already made an important contribution to our knowledge of important conditions for learning from errors. We would like to encourage future works on learning from errors that build on these insights and integrate them with findings from research on erroneous examples and formative interactive feedback strategies. By doing so, further insights into the interplay of core components that might contribute to a supportive error climate (see Figure 1) will be gained.

## AUTHOR CONTRIBUTIONS

**Susanne Narciss:** Conceptualization; supervision; writing – original draft; writing – review and editing; funding acquisition; investigation. **Ecenaz Alemdag:** Conceptualization; visualization; writing – original draft; writing – review and editing; investigation; supervision.

## CONFLICT OF INTEREST STATEMENT

We have no conflicts of interest to disclose.

## Data Availability

Data sharing is not applicable to this article as no new data were created or analyzed in this study.

## References

[bjep12716-bib-0001] Adams, D. M. , McLaren, B. M. , Durkin, K. , Mayer, R. E. , Rittle‐Johnson, B. , Isotani, S. , & van Velsen, M. (2014). Using erroneous examples to improve mathematics learning with a web‐based tutoring system. Computers in Human Behavior, 36, 401–411. 10.1016/j.chb.2014.03.053

[bjep12716-bib-0002] Ajzen, I. (1991). The theory of planned behavior. Organizational Behavior and Human Decision Processes, 50(2), 179–211. 10.1016/0749-5978(91)90020-T

[bjep12716-bib-0003] Alemdag, E. , & Yildirim, Z. (2022a). Design and development of an online formative peer assessment environment with instructional scaffolds. Educational Technology Research and Development, 70, 1359–1389. 10.1007/s11423-022-10115-x

[bjep12716-bib-0004] Alemdag, E. , & Yildirim, Z. (2022b). Effectiveness of online regulation scaffolds on peer feedback provision and uptake: A mixed methods study. Computers & Education, 188, 104574. 10.1016/j.compedu.2022.104574

[bjep12716-bib-0005] Alexander, R. J. (2008). Towards dialogic teaching: Rethinking classroom talk (4th ed.). Dialogos.

[bjep12716-bib-0006] Allal, L. (2016). The co‐regulation of student learning in an assessment for learning culture. In L. Allal & D. Laveault (Eds.), Assessment for learning: Meeting the challenge of implementation (pp. 259–273). Springer.

[bjep12716-bib-0007] Alqassab, M. , Strijbos, J. W. , & Ufer, S. (2018). Training peer‐feedback skills on geometric construction tasks: Role of domain knowledge and peer‐feedback levels. European Journal of Psychology of Education, 33(1), 11–30. 10.1007/s10212-017-0342-0

[bjep12716-bib-0008] Bannert, M. (2009). Promoting self‐regulated learning through prompts. Zeitschrift für Pädagogische Psychologie, 23(2), 139–145. 10.1024/1010-0652.23.2.139

[bjep12716-bib-0009] Barbieri, C. A. , & Booth, J. L. (2020). Mistakes on display: Incorrect examples refine equation solving and algebraic feature knowledge. Applied Cognitive Psychology, 34(4), 862–878. 10.1002/acp.3663

[bjep12716-bib-0010] Barbieri, C. A. , Miller‐Cotto, D. , Clerjuste, S. N. , & Chawla, K. (2023). A meta‐analysis of the worked examples effect on mathematics performance. Educational Psychology Review, 35(1), 1–33. 10.1007/s10648-023-09745-1

[bjep12716-bib-0011] Beege, M. , Schneider, S. , Nebel, S. , Zimm, J. , Windisch, S. , & Rey, G. D. (2021). Learning programming from erroneous worked‐examples. Which type of error is beneficial for learning? Learning and Instruction, 75, 101497. 10.1016/j.learninstruc.2021.101497

[bjep12716-bib-0012] Butterfield, B. , & Metcalfe, J. (2001). Errors committed with high confidence are hypercorrected. Journal of Experimental Psychology: Learning, Memory, and Cognition, 27(6), 1491–1494. 10.1037//0278-7393.27.6.1491 11713883

[bjep12716-bib-0013] Carless, D. , & Boud, D. (2018). The development of student feedback literacy: Enabling uptake of feedback. Assessment & Evaluation in Higher Education, 43(8), 1315–1325. 10.1080/02602938.2018.1463354

[bjep12716-bib-0014] Carless, D. , & Winstone, N. (2023). Teacher feedback literacy and its interplay with student feedback literacy. Teaching in Higher Education, 28(1), 150–163. 10.1080/13562517.2020.1782372

[bjep12716-bib-0015] Chen, T. (2016). Technology‐supported peer feedback in ESL/EFL writing classes: A research synthesis. Computer Assisted Language Learning, 29(2), 365–397. 10.1080/09588221.2014.960942

[bjep12716-bib-0016] Covington, M. (2009). Self‐worth theory: Retrospection and prospects. In K. R. Wentzel & A. Wigfield (Eds.), Handbook of motivation at school (pp. 155–184). Routledge.

[bjep12716-bib-0094] DeLiema, D. , Hufnagle, A. , & Ovies‐Bocanegra, M. (2024). Contrasting stances at the crossroads of debugging learning opportunities. The British Journal of Educational Psychology. 10.1111/bjep.12666 PMC11802966 38366305

[bjep12716-bib-0017] Darabi, A. , Arrington, T. L. , & Sayilir, E. (2018). Learning from failure: A meta‐analysis of the empirical studies. Educational Technology Research and Development, 66, 1101–1118. 10.1007/s11423-018-9579-9

[bjep12716-bib-0097] Di Battista, S. (2024). ‘She is failing; he is learning’: Gender‐differentiated attributions for girls’ and boys’ errors. The British Journal of Educational Psychology. 10.1111/bjep.12665 38369383

[bjep12716-bib-0103] Dresel, M. , Daumiller, M. , Spear, J. , Janke, S. , Dickhäuser, O. , & Steuer, G. (2024). Learning from errors in mathematics classrooms: Development over 2 years in dependence of perceived error climate. The British Journal of Educational Psychology. 10.1111/bjep.12697 PMC11802964 38888062

[bjep12716-bib-0018] Ge, X. , & Land, S. M. (2004). A conceptual framework for scaffolding III‐structured problem‐solving processes using question prompts and peer interactions. Educational Technology Research and Development, 52(2), 5–22. 10.1007/BF02504836

[bjep12716-bib-0019] Grassinger, R. , Scheunpflug, A. , Zeinz, H. , & Dresel, M. (2018). Smart is who makes lots of errors? The relevance of adaptive reactions to errors and a positive error climate for academic achievement. High Ability Studies, 29(1), 37–49. 10.1080/13598139.2018.1459294

[bjep12716-bib-0020] Grolnick, W. S. , & Ryan, R. M. (1989). Parent styles associated with children's self‐regulation and competence in school. Journal of Educational Psychology, 81(2), 143–154. 10.1037/0022-0663.81.2.143

[bjep12716-bib-0022] Haimovitz, K. , & Dweck, C. S. (2016). Parents' views of failure predict children's fixed and growth intelligence mind‐sets. Psychological Science, 27(6), 859–869. 10.1177/0956797616639727 27113733

[bjep12716-bib-0024] Kapur, M. (2010). Productive failure in mathematical problem solving. Instructional Science, 38, 523–550. 10.1007/s11251-009-9093-x

[bjep12716-bib-0025] Kapur, M. (2014a). Comparing learning from productive failure and vicarious failure. Journal of the Learning Sciences, 23(4), 651–677. 10.1080/10508406.2013.819000

[bjep12716-bib-0026] Kapur, M. (2014b). Productive failure in learning math. Cognitive Science, 38(5), 1008–1022. 10.1111/cogs.12107 24628487

[bjep12716-bib-0027] Khasawneh, A. A. , Al‐Barakat, A. A. , & Almahmoud, S. A. (2022). The effect of error analysis‐based learning on proportional reasoning ability of seventh‐grade students. *Frontiers* . Education, 7, Article 899288. 10.3389/feduc.2022.899288

[bjep12716-bib-0028] Kim, M. , & Ryu, J. (2013). The development and implementation of a web‐based formative peer assessment system for enhancing students' metacognitive awareness and performance in ill‐structured tasks. Educational Technology Research and Development, 61(4), 549–561. 10.1007/s11423-012-9266-1

[bjep12716-bib-0029] Klopp, E. , Stark, R. , Kopp, V. , & Fischer, M. R. (2013). Psychological factors affecting medical students' learning with erroneous worked examples. Journal of Education and Learning, 2(1), 158–170. 10.5539/jel.v2n1p158

[bjep12716-bib-0030] Laudel, H. , & Narciss, S. (2023). The effects of internal feedback and self‐compassion on the perception of negative feedback and post‐feedback learning behavior. Studies in Educational Evaluation, 77, 101237. 10.1016/j.stueduc.2023.101237

[bjep12716-bib-0031] Lauzier, M. , & Bilodeau Clarke, A. (2023). Linking learning goal orientation to learning from error: The mediating role of motivation to learn and metacognition. European Journal of Training and Development, 48, 485–500. 10.1108/EJTD-11-2022-0127

[bjep12716-bib-0032] Li, H. , Xiong, Y. , Hunter, C. V. , Guo, X. , & Tywoniw, R. (2020). Does peer assessment promote student learning? A meta‐analysis. Assessment & Evaluation in Higher Education, 45(2), 193–211. 10.1080/02602938.2019.1620679

[bjep12716-bib-0033] Li, L. (2017). The role of anonymity in peer assessment. Assessment & Evaluation in Higher Education, 42(4), 645–656. 10.1080/02602938.2016.1174766

[bjep12716-bib-0034] Liu, S. , Liu, S. , Liu, Z. , Peng, X. , & Yang, Z. (2022). Automated detection of emotional and cognitive engagement in MOOC discussions to predict learning achievement. Computers & Education, 181, 104461. 10.1016/j.compedu.2022.104461

[bjep12716-bib-0035] Luyten, P. , Fontaine, J. R. , & Corveleyn, J. (2002). Does the test of self‐conscious affect (TOSCA) measure maladaptive aspects of guilt and adaptive aspects of shame? An empirical investigation. Personality and Individual Differences, 33(8), 1373–1387. 10.1016/S0191-8869(02)00197-6

[bjep12716-bib-0036] McLaren, B. M. , Adams, D. M. , & Mayer, R. E. (2015). Delayed learning effects with erroneous examples: A study of learning decimals with a web‐based tutor. International Journal of Artificial Intelligence in Education, 25(4), 520–542. 10.1007/s40593-015-0064-x

[bjep12716-bib-0037] Mera, Y. , Rodríguez, G. , & Marin‐Garcia, E. (2022). Unraveling the benefits of experiencing errors during learning: Definition, modulating factors, and explanatory theories. Psychonomic Bulletin & Review, 1–13, 753–765. 10.3758/s13423-021-02022-8 34820785

[bjep12716-bib-0038] Metcalfe, J. (2017). Learning from errors. Annual Review of Psychology, 68, 465–489. 10.1146/annurev-psych-010416-044022 27648988

[bjep12716-bib-0096] Metcalfe, J. , Xu, J. , Vuorre, M. , Siegler, R. , Wiliam, D. , & Bjork, R. A. (2024). Learning from errors versus explicit instruction in preparation for a test that counts. The British Journal of Educational Psychology. 10.1111/bjep.12651 38212139

[bjep12716-bib-0039] Meyer, A. , Carlton, C. , Chong, L. J. , & Wissemann, K. (2019). The presence of a controlling parent is related to an increase in the error‐related negativity in 5–7 year‐old children. Journal of Abnormal Child Psychology, 47, 935–945. 10.1007/s10802-018-0503-x 30610550

[bjep12716-bib-0040] Narciss, S. (2008). Feedback strategies for interactive learning tasks. In J. M. Spector , M. D. Merrill , J. G. van Merrienboer , & M. P. Driscoll (Eds.), Handbook of research on educational communications and technology (3rd ed., pp. 125–143). Lawrence Erlbaum Associates.

[bjep12716-bib-0041] Narciss, S. (2013). Designing and evaluating tutoring feedback strategies for digital learning environments on the basis of the interactive tutoring feedback model. Digital Education Review, 23(1), 7–26.

[bjep12716-bib-0042] Narciss, S. (2017). Conditions and effects of feedback viewed through the lens of the interactive tutoring feedback model. In D. Carless , S. M. Bridges , C. K. Y. Chan , & R. Glofcheski (Eds.), Scaling up assessment for learning in higher education (pp. 173–189). Springer Singapore.

[bjep12716-bib-0043] Narciss, S. , Prescher, C. , Khalifah, L. , & Körndle, H. (2022). Providing external feedback and prompting the generation of internal feedback fosters achievement, strategies and motivation in concept learning. Learning and Instruction, 82, 101658. 10.1016/j.learninstruc.2022.101658

[bjep12716-bib-0044] Narciss, S. , Sosnovsky, S. , Schnaubert, L. , Andrès, E. , Eichelmann, A. , Goguadze, G. , & Melis, E. (2014). Exploring feedback and student characteristics relevant for personalizing feedback strategies. Computers & Education, 71, 56–76. 10.1016/j.compedu.2013.09.011

[bjep12716-bib-0045] Ng, F. Y. , Kenney‐Benson, G. A. , & Pomerantz, E. M. (2004). Children's achievement moderates the effects of mothers' use of control and autonomy support. Child Development, 75(3), 764–780. 10.1111/j.1467-8624.2004.00705.x 15144485

[bjep12716-bib-0046] Nicol, D. , & McCallum, S. (2022). Making internal feedback explicit: Exploiting the multiple comparisons that occur during peer review. Assessment & Evaluation in Higher Education, 47(3), 424–443. 10.1080/02602938.2021.1924620

[bjep12716-bib-0047] Norman, D. A. (1981). Categorization of action slips. Psychological Review, 88(1), 1–15. 10.1037/0033-295X.88.1.1

[bjep12716-bib-0048] Ohlsson, S. (1996a). Learning from error and the design of task environments. International Journal of Educational Research, 25(5), 419–448. 10.1016/S0883-0355(97)81236-0

[bjep12716-bib-0049] Ohlsson, S. (1996b). Learning from performance errors. Psychological Review, 103(2), 241–262. 10.1037/0033-295X.103.2.241

[bjep12716-bib-0050] Oser, F. , & Spychiger, M. (2005). Lernen ist schmerzhaft. Zur Theorie des negativen Wissens und zur praxis der Fehlerkultur [learning hurts. From a theory of negative knowledge towards practicing an error culture]. Beltz.

[bjep12716-bib-0051] Panadero, E. , & Jonsson, A. (2013). The use of scoring rubrics for formative assessment purposes revisited: A review. Educational Research Review, 9, 129–144. 10.1016/j.edurev.2013.01.002

[bjep12716-bib-0052] Panadero, E. , Jonsson, A. , Pinedo, L. , & Fernández‐Castilla, B. (2023). Effects of rubrics on academic performance, self‐regulated learning, and self‐efficacy: A meta‐analytic review. Educational Psychology Review, 35(4), 113. 10.1007/s10648-023-09823-4

[bjep12716-bib-0053] Pekrun, R. (2006). The control‐value theory of achievement emotions: Assumptions, corollaries, and implications for educational research and practice. Educational Psychology Review, 18, 315–341. 10.1007/s10648-006-9029-9

[bjep12716-bib-0054] Pekrun, R. , Marsh, H. W. , Elliot, A. J. , Stockinger, K. , Perry, R. P. , Vogl, E. , Goetz, T. , van Tilburg, W. A. P. , Lüdtke, O. , & Vispoel, W. P. (2023). A three‐dimensional taxonomy of achievement emotions. Journal of Personality and Social Psychology, 124(1), 145–178. 10.1037/pspp0000448 36521161

[bjep12716-bib-0102] Peterson, E. R. , Sharma, T. , Bird, A. , Henderson, A. M. E. , Ramgopal, V. , Reese, E. , & Morton, S. M. B. (2024). How mothers talk to their children about failure, mistakes and setbacks is related to their children's fear of failure. The British Journal of Educational Psychology. 10.1111/bjep.12685 PMC11802963 38693065

[bjep12716-bib-0055] Puntambekar, S. (2022). Distributed scaffolding: Scaffolding students in classroom environments. Educational Psychology Review, 34(1), 451–472. 10.1007/s10648-021-09636-3

[bjep12716-bib-0056] Reason, J. (1990). Human error. Cambridge University Press.

[bjep12716-bib-0057] Ryan, R. M. , & Deci, E. L. (2017). Self‐determination theory: Basic psychological needs in motivation, development, and wellness. Guilford press.

[bjep12716-bib-0058] Safadi, R. (2022). Supporting student learning from diagnosing erroneous examples when contrasting them with worked examples in the physics classroom. International Journal of Science Education, 44(2), 245–270. 10.1080/09500693.2021.2023834

[bjep12716-bib-0059] Safadi, R. , & Yerushalmi, E. (2014). Problem solving vs. troubleshooting tasks: The case of sixth‐grade students studying simple electric circuits. International Journal of Science and Mathematics Education, 12, 1341–1366. 10.1007/s10763-013-9461-5

[bjep12716-bib-0060] Sasou, K. , & Reason, J. (1999). Team errors: Definition and taxonomy. Reliability Engineering & System Safety, 65(1), 1–9.

[bjep12716-bib-0101] Schmid, R. , Smit, R. , Robin, N. , & Strahl, A. (2024). The role of momentary emotions in promoting error learning orientation among lower secondary school students: An intervention study embedded in a short visual programming course. The British Journal of Educational Psychology. 10.1111/bjep.12681 PMC11802965 38503561

[bjep12716-bib-0061] Seifried, J. , Wuttke, E. , Türling, J. M. , Krille, C. , & Paul, O. (2015). Teachers' strategies for handling student errors–the contribution of teacher training programs. In M. Gartmeier , H. Gruber , T. Hascher , & H. Heid (Eds.), Fehler–Ihre Funktion im Kontext individueller und gesellschaftlicher Entwicklung (pp. 177–188). Waxmann.

[bjep12716-bib-0100] Sharabi, Y. , & Roth, G. (2024). Emotion regulation styles and the tendency to learn from academic failures. The British Journal of Educational Psychology. 10.1111/bjep.12696 PMC11802962 38877349

[bjep12716-bib-0062] Sharot, T. , Rollwage, M. , Sunstein, C. R. , & Fleming, S. M. (2023). Why and when beliefs change. Perspectives on Psychological Science, 18(1), 142–151. 10.1177/17456916221082 35939828

[bjep12716-bib-0063] Shih, S. C. , Chang, C. C. , Kuo, B. C. , & Huang, Y. H. (2023). Mathematics intelligent tutoring system for learning multiplication and division of fractions based on diagnostic teaching. Education and Information Technologies, 28(7), 9189–9210. 10.1007/s10639-022-11553-z PMC9831028 36643382

[bjep12716-bib-0064] Siegler, R. S. (2002). Microgenetic studies of self‐explanations. In N. Granott & J. Parziale (Eds.), Microdevelopment: Transition processes in development and learning (pp. 31–58). Cambridge University.

[bjep12716-bib-0065] Siemer, J. , & Angelides, M. C. (1998). Towards an intelligent tutoring system architecture that supports remedial tutoring. Artificial Intelligence Review, 12, 469–511. 10.1023/A:1006588626632

[bjep12716-bib-0095] Simpson, A. , Anderson, A. , Goeke, M. , Caruana, D. , & Maltese, A. V. (2023). Identifying and shifting educators’ failure pedagogical mindsets through reflective practices. The British Journal of Educational Psychology. 10.1111/bjep.12658 38140824

[bjep12716-bib-0066] Simpson, A. , Maltese, A. V. , Anderson, A. , & Sung, E. (2020). Failures, errors, and mistakes: A systematic review of the literature. In E. Vanderheide & C.‐H. Mayer (Eds.), Mistakes, errors and failures across cultures: Navigating potentials (pp. 347–362). Springer. 10.1007/978-3-030-35574-6_18

[bjep12716-bib-0067] Sitzman, D. M. , Rhodes, M. G. , & Tauber, S. K. (2014). Prior knowledge is more predictive of error correction than subjective confidence. Memory & Cognition, 42, 84–96. 10.3758/s13421-013-0344-3 23797971

[bjep12716-bib-0068] Sitzman, D. M. , Rhodes, M. G. , Tauber, S. K. , & Liceralde, V. R. T. (2015). The role of prior knowledge in error correction for younger and older adults. Aging, Neuropsychology, and Cognition, 22(4), 502–516. 10.1080/13825585.2014.993302 25558782

[bjep12716-bib-0098] Soncini, A. , Matteucci, M. C. , Tomasetto, C. , & Butera, F. (2024). Supportive error feedback fosters students’ adaptive reactions towards errors: Evidence from a targeted online intervention with Italian middle school students. The British Journal of Educational Psychology. 10.1111/bjep.12679 38499362

[bjep12716-bib-0069] Soncini, A. , Visintin, E. P. , Matteucci, M. C. , Tomasetto, C. , & Butera, F. (2022). Positive error climate promotes learning outcomes through students' adaptive reactions towards errors. Learning and Instruction, 80, 101627. 10.1016/j.learninstruc.2022.101627

[bjep12716-bib-0070] Steuer, G. , & Dresel, M. (2015). A constructive error climate as an element of effective learning environments. Psychological Test and Assessment Modeling, 57(2), 262–275.

[bjep12716-bib-0093] Steuer, G. , Grecu, A. L. , & Mori, J. (2024). Error climate and alienation from teachers: A longitudinal analysis in primary school. The British Journal of Educational Psychology. 10.1111/bjep.12659 PMC11802967 38168019

[bjep12716-bib-0071] Steuer, G. , Rosentritt‐Brunn, G. , & Dresel, M. (2013). Dealing with errors in mathematics classrooms: Structure and relevance of perceived error climate. Contemporary Educational Psychology, 38(3), 196–210. 10.1016/j.cedpsych.2013.03.002

[bjep12716-bib-0072] Theobald, M. , Colantonio, J. , Bascandziev, I. , Bonawitz, E. , & Brod, G. (2024). Do reflection prompts promote children's conflict monitoring and revision of misconceptions? Child Development, 95, e253–e269. 10.1111/cdev.14081 38366838

[bjep12716-bib-0073] Tjosvold, D. , Yu, Z. Y. , & Hui, C. (2004). Team learning from mistakes: The contribution of cooperative goals and problem‐solving. Journal of Management Studies, 41(7), 1223–1245. 10.1111/j.1467-6486.2004.00473.x

[bjep12716-bib-0074] Tulis, M. , & Ainley, M. (2011). Interest, enjoyment and pride after failure experiences? Predictors of students' state‐emotions after success and failure during learning in mathematics. Educational Psychology, 31(7), 779–807. 10.1080/01443410.2011.608524

[bjep12716-bib-0099] Tulis, M. , & Dresel, M. (2024). Effects on and consequences of responses to errors: Results from two experimental studies. The British Journal of Educational Psychology. 10.1111/bjep.12686 PMC11802961 38719784

[bjep12716-bib-0075] Tulis, M. , Steuer, G. , & Dresel, M. (2016). Learning from errors: A model of individual processes. Frontline Learning Research, 4(2), 12–26. 10.14786/flr.v4i2.168

[bjep12716-bib-0076] van Gog, T. , Rummel, N. , & Renkl, A. (2019). Learning how to solve problems by studying examples. In J. Dunlosky & K. A. Rawson (Eds.), The Cambridge handbook of cognition and education (pp. 183–208). Cambridge University Press.

[bjep12716-bib-0077] VanLehn, K. (1988). Toward a theory of impasse‐driven learning. In H. Mandl & A. Lesgold (Eds.), Learning issues for intelligent tutoring systems (pp. 19–41). Springer.

[bjep12716-bib-0078] VanLehn, K. (1999). Rule‐learning events in the acquisition of a complex skill: An evaluation of cascade. The Journal of the Learning Sciences, 8(1), 71–125. 10.1207/s15327809jls0801_3

[bjep12716-bib-0079] Vogl, E. , Pekrun, R. , Murayama, K. , & Loderer, K. (2020). Surprised–curious–confused: Epistemic emotions and knowledge exploration. Emotion, 20(4), 625–641. 10.1037/emo0000578 30883147

[bjep12716-bib-0080] Watson, S. , Gomez, R. , & Gullone, E. (2017). The shame and guilt scales of the test of self‐conscious affect–adolescent (TOSCA‐A): Factor structure, concurrent and discriminant validity, and measurement and structural invariance across ratings of males and females. Assessment, 24(4), 517–527. 10.1177/1073191115608942 26450945

[bjep12716-bib-0081] Watson, S. D. , Gomez, R. , & Gullone, E. (2016). The shame and guilt scales of the test of self‐conscious affect‐adolescent (TOSCA‐A): Psychometric properties for responses from children, and measurement invariance across children and adolescents. Frontiers in Psychology, 7, 635. 10.3389/fpsyg.2016.00635 27242573 PMC4860388

[bjep12716-bib-0082] Wong, S. S. H. (2023). Deliberate erring improves far transfer of learning more than errorless elaboration and spotting and correcting others' errors. Educational Psychology Review, 35(1), 16. 10.1007/s10648-023-09739-z 36776579 PMC9902256

[bjep12716-bib-0083] Wong, S. S. H. , & Lim, S. W. H. (2022a). Deliberate errors promote meaningful learning. Journal of Educational Psychology, 114(8), 1817–1831. 10.1037/edu0000720

[bjep12716-bib-0084] Wong, S. S. H. , & Lim, S. W. H. (2022b). The derring effect: Deliberate errors enhance learning. Journal of Experimental Psychology: General, 151(1), 25–40. 10.1037/xge0001072 34242048

[bjep12716-bib-0085] Wood, J. (2021). A dialogic technology‐mediated model of feedback uptake and literacy. Assessment & Evaluation in Higher Education, 46(8), 1173–1190. 10.1080/02602938.2020.1852174

[bjep12716-bib-0086] Wuttke, E. , & Seifried, J. (2017). Competence, teacher competence and professional error competence: An introduction. In E. Wuttke & J. Seifried (Eds.), Professional error competence of preservice teachers: Evaluation and support (pp. 1–14). Springer.

[bjep12716-bib-0087] Yang, Z. , Wang, M. , Cheng, H. N. H. , Liu, S. , Liu, L. , & Chan, T.‐W. (2016). The effects of learning from correct and erroneous examples in individual and collaborative settings. The Asia‐Pacific Education Researcher, 25(2), 219–227. 10.1007/s40299-015-0253-2

[bjep12716-bib-0092] Zhang, Q. , & Fiorella, L. (2023). An integrated model of learning from errors. Educational Psychologist, 58(1), 18–34. 10.1080/00461520.2022.2149525

[bjep12716-bib-0088] Zhang, Y. , Paquette, L. , Baker, R. S. , Ocumpaugh, J. , Bosch, N. , Biswas, G. , & Munshi, A. (2021). Can strategic behaviour facilitate confusion resolution? The interplay between confusion and metacognitive strategies in Betty's brain. Journal of Learning Analytics, 8(3), 28–44.

[bjep12716-bib-0089] Zheng, L. , Cui, P. , Li, X. , & Huang, R. (2018). Synchronous discussion between assessors and assessees in web‐based peer assessment: Impact on writing performance, feedback quality, meta‐cognitive awareness and self‐efficacy. Assessment & Evaluation in Higher Education, 43(3), 500–514. 10.1080/02602938.2017.1370533

[bjep12716-bib-0090] Zhu, Q. , & Carless, D. (2018). Dialogue within peer feedback processes: Clarification and negotiation of meaning. Higher Education Research and Development, 37(4), 883–897. 10.1080/07294360.2018.1446417

[bjep12716-bib-0091] Zhu, Q. , & To, J . (2022). Proactive receiver roles in peer feedback dialogue: Facilitating receivers' self‐regulation and co‐regulating providers' learning. Assessment & Evaluation in Higher Education, 47(8), 1200–1212. 10.1080/02602938.2021.2017403

